# Elevated Doppler-estimated right ventricular systolic pressure in complete atrioventricular block and its response to atrioventricular-synchronous pacing

**DOI:** 10.3389/fcvm.2026.1967085

**Published:** 2026-10-28

**Authors:** Byeng-Ju Son, Sungyun Jung, Byung-Jun Kim, Hong-Ju Kim, Hyongjun Kim

**Affiliations:** Division of Cardiology, Department of Internal Medicine, Dongkang Medical Center, Ulsan, Republic of Korea

**Keywords:** atrioventricular synchrony, cardiac pacing, complete atrioventricular block, Doppler echocardiography, pulmonary artery pressure, right ventricular systolic pressure, tricuspid regurgitation

## Abstract

**Background:**

Complete atrioventricular block causes both bradycardia and loss of atrioventricular synchrony. Case reports have described marked elevations in Doppler-estimated right ventricular systolic pressure that fall after pacing, but the prevalence and consistency of this phenotype have not been examined in a cohort.

**Methods:**

We retrospectively studied consecutive patients in sinus rhythm with complete atrioventricular block and a left ventricular ejection fraction of at least 50%, identified from 142 permanent pacemaker implantations at one center between 2021 and 2026. Right atrial pressure was estimated using current guideline categories. The prespecified primary endpoint was the paired change in Doppler-estimated right ventricular systolic pressure between the native state and the first study within 72 h of atrioventricular-synchronous pacing. Tricuspid regurgitant signal quality was graded on envelope completeness and analyzed alongside it.

**Results:**

Thirty patients formed the native cohort and 14 had paired studies. Doppler-estimated right ventricular systolic pressure was 44.0 mmHg (interquartile range 32.4–50.3) during native block and was 35 mmHg or greater in 21 patients. In the paired cohort it fell from 46.6 to 25.0 mmHg in all 14 patients (median relative change −51.4%; *P* < 0.001), and the proportion with values of 35 mmHg or greater fell from 11 to 3 (*P* = 0.008). Both the tricuspid regurgitant gradient and the estimated right atrial pressure decreased, and tricuspid regurgitation grade fell in 7 patients and increased in none. Paced QRS duration increased, and the heart-rate change was not correlated with the change in Doppler-estimated right ventricular systolic pressure. Signal quality tended to decline after pacing; in a *post hoc* analysis restricted to the 7 patients in whom it was unchanged or improved, the reduction persisted (median relative change −33.8%; *P* = 0.016). In four catheterized patients, Doppler estimates diverged from invasive pressures in both directions.

**Conclusion:**

Elevated Doppler-estimated right ventricular systolic pressure was common during complete atrioventricular block and fell consistently after atrioventricular-synchronous pacing. Because signal quality changed concurrently and invasive pressure did not invariably track the estimate, the magnitude of the observed Doppler reduction, and whether pulmonary artery pressure itself falls, remain uncertain.

## 1 Introduction

In complete atrioventricular block the atria and ventricles depolarize independently, and two things happen at once. The ventricular rate falls to that of a subsidiary escape pacemaker, and the fixed temporal relation between atrial and ventricular contraction is lost. The hemodynamic cost of the second of these has been recognized since the earliest pacing studies, in which atrial-triggered pacing produced a higher cardiac output and lower left ventricular filling pressures at rest than rate-matched fixed-rate ventricular pacing (1). The same dependence has been shown in contemporary within-patient comparisons, in which stroke volume was higher during atrioventricular-synchronous than during atrioventricular-asynchronous pacing in every patient studied (2). Atrial contraction occurring against closed atrioventricular valves generates cannon a waves and abrupt elevations in atrial and systemic venous pressure (3). Most of this work, and the pacemaker syndrome literature that followed it, has concerned systemic hemodynamics (4).

The consequences for the right heart and the pulmonary circulation are far less well characterized. Isolated case reports have described striking elevations in Doppler-estimated right ventricular systolic pressure during untreated complete heart block that fell substantially after permanent pacing (5, 6). The proposed explanations have differed: one report attributed the finding chiefly to the augmented right-sided stroke volume that accompanies bradycardia (5), whereas a recent report attributed it to atrioventricular dyssynchrony itself (6). Whether this phenotype is exceptional or common, whether the direction of change after pacing is consistent between patients, and how rapidly that change occurs have not, to our knowledge, been systematically examined in a cohort. The question is not only mechanistic. An isolated elevation in estimated pulmonary pressure discovered during untreated complete atrioventricular block may prompt referral for evaluation of pulmonary vascular disease, and the appropriate interpretation depends on whether the finding is likely to persist after rhythm and pacing status have stabilized.

Interpretation is further complicated by the nature of the measurement. Doppler estimation of pulmonary artery pressure has wide limits of agreement against invasive measurement, with substantial errors in both directions even when the two are obtained simultaneously (7–9). Complete atrioventricular dissociation adds a specific difficulty, because ventricular filling varies from beat to beat, so that the beat selected for measurement and the completeness of the regurgitant envelope both influence the result. The extent to which rhythm and pacing state influence Doppler–invasive agreement remains uncertain.

We therefore examined a consecutive single-center cohort of patients in sinus rhythm with complete atrioventricular block who underwent permanent pacemaker implantation. Our aims were to determine how often an elevated Doppler-estimated right ventricular systolic pressure was present during native complete atrioventricular block, to quantify its change within 72 h of atrioventricular-synchronous pacing in patients with paired studies, and to examine that change alongside the change in tricuspid regurgitant signal quality. In a small invasive substudy and in two patients studied serially across pacing states, we also descriptively compared Doppler estimates with invasive pulmonary artery pressures.

## 2 Materials and methods

### 2.1 Study design and population

This was a retrospective, single-center, observational study conducted at Dongkang Medical Center, Ulsan, Republic of Korea. The study was approved by the institutional review board (approval number Dongkang 2026-06-05), which waived the requirement for written informed consent because the analysis used data generated during routine clinical care.

Records were reviewed through June 2026. We identified all patients who underwent permanent pacemaker implantation at our institution between January 2021 and May 2026; of 142 consecutive implantations, 59 were performed for complete atrioventricular block. Patients were eligible if they were in sinus rhythm with complete atrioventricular block, had a left ventricular ejection fraction of at least 50%, and had an interpretable transthoracic echocardiogram before implantation.

Complete atrioventricular block was defined on the 12-lead electrocardiogram as complete atrioventricular dissociation with an atrial rate exceeding the ventricular rate, a regular escape rhythm, and no conducted beats; recordings in which any conducted beat was present were classified as high-grade atrioventricular block and excluded. Dissociation was cross-checked against the transmitral inflow pattern on the corresponding echocardiogram, in which dissociated A waves recurred at intervals unrelated to ventricular diastole. All electrocardiograms were interpreted by the same investigator who performed the echocardiographic measurements, with ambiguous tracings adjudicated in consultation with an electrophysiologist.

Twenty-four patients were excluded: atrial fibrillation (*n* = 5), paroxysmal complete block or recovery of atrioventricular conduction (*n* = 7), no pre-implantation echocardiogram (*n* = 4), left ventricular ejection fraction below 50% (*n* = 2), an aortic valve area below 1.5 cm^2^ by planimetry (*n* = 3), atrial septal defect (*n* = 1), and device-related exclusions comprising lead exposure and pocket infection (*n* = 2). Of the 35 remaining patients, five were already receiving temporary VVI pacing at the time of the baseline echocardiogram and were excluded from quantitative analyses because no unpaced reference study was available. The native cohort therefore comprised 30 patients.

Fourteen of these patients underwent repeat echocardiography within 72 h of atrioventricular-synchronous pacing and formed the primary paired cohort. Post-implantation echocardiography was not mandated by protocol and was obtained at the discretion of the treating physician.

#### 2.1.1 Endpoints

The prespecified primary endpoint was the change in Doppler-estimated right ventricular systolic pressure between the native complete atrioventricular block state and the first echocardiogram obtained after atrioventricular-synchronous pacing, assessed in the paired cohort. Prespecified secondary endpoints were the prevalence of an elevated Doppler-estimated right ventricular systolic pressure during native complete atrioventricular block, and the paired changes in peak tricuspid regurgitant velocity, tricuspid regurgitant pressure gradient, estimated right atrial pressure, inferior vena cava diameter, and tricuspid regurgitation grade. All remaining analyses, including those involving the RR/PP ratio, left ventricular outflow-tract flow measurements, and E/e′, were exploratory.

### 2.2 Rhythm comparators and invasive substudy

Right heart catheterization was performed in four patients on clinical grounds. Two were in sinus rhythm and belonged to the native cohort; two had atrial fibrillation with complete atrioventricular block and are presented as descriptive rhythm comparators outside the native cohort. Because only two cases were available in each rhythm group and catheterization was clinically selected rather than protocol-driven, no formal between-rhythm statistical comparison was performed.

Two patients underwent serial echocardiography across native rhythm, temporary VVI pacing, and atrioventricular-synchronous pacing. These serial observations are presented descriptively and are not treated as a formal comparison between pacing modes.

### 2.3 Definition of atrioventricular-synchronous pacing

Atrioventricular-synchronous pacing comprised DDD and VDD modes and was delivered by a transvenous dual-chamber system in 27 patients, a leadless dual-chamber system with communicating right atrial and right ventricular components (Aveir DR, Abbott, Abbott Park, IL, USA) in 2, and a single-component leadless device providing VDD pacing (Micra AV, Medtronic, Minneapolis, MN, USA) in 1. Atrial tracking was confirmed on the post-implantation electrocardiogram in all 30 patients by a consistent *P*-to-paced-QRS interval, and additionally on transmitral Doppler by a fixed E–A sequence in every patient who underwent post-implantation echocardiography.

### 2.4 Echocardiography

Studies were performed using one of five commercially available systems: iE33 or EPIQ CVx (Philips Healthcare, Andover, MA, USA), or Vivid E95, Vivid E90, or Vivid iq (GE HealthCare, Horten, Norway). The Vivid iq is a portable system and was used for bedside studies when transfer to the echocardiography laboratory was not feasible. The system used was determined by clinical circumstance and equipment availability rather than by protocol.

Although the clinical studies had originally been reported by several echocardiographers, all images were retrieved and re-analyzed for this study by a single investigator, who identified the tricuspid regurgitant envelope, selected the beat for measurement, assigned the signal-quality grade, and estimated right atrial pressure from the inferior vena cava. The original clinical reports were used only to identify eligible studies and did not contribute measurements to the analysis. Because the pacing state is evident from the images and the electrocardiographic tracing, blinding to pacing status was not feasible.

#### 2.4.1 Tricuspid regurgitant velocity and right ventricular systolic pressure

Peak tricuspid regurgitant velocity was measured by continuous-wave Doppler from the view in which the regurgitant jet was best profiled. Because ventricular filling varies from beat to beat when atrial and ventricular activity are dissociated, beats were first screened for the most complete envelope with an identifiable peak within each recording, and the highest velocity among those beats was taken. The same rule was applied to every recording regardless of its overall signal quality. After atrioventricular-synchronous pacing the Doppler profiles were substantially more uniform and little beat selection was generally required.

The tricuspid regurgitant pressure gradient was calculated from the simplified Bernoulli equation as four times the square of the peak velocity, the approach originally validated against catheterization for the noninvasive estimation of right ventricular systolic pressure (10, 11). Right atrial pressure was estimated from inferior vena cava diameter and respirophasic collapse in accordance with the current recommendations of the American Society of Echocardiography (12), using a diameter cutoff of 2.1 cm and 50% inspiratory collapse. An inferior vena cava of 2.1 cm or less that collapsed by more than 50% was assigned 3 mmHg, and an inferior vena cava exceeding 2.1 cm that collapsed by less than 50% was assigned 15 mmHg. The two discordant patterns — a normal-sized vessel that did not collapse and a dilated vessel that did — were classified as indeterminate and assigned the intermediate value of 8 mmHg, following the guideline's provision for cases in which the secondary indices recommended for refining an indeterminate estimate are unavailable. Because the study was retrospective, those secondary indices — right atrial enlargement, interatrial septal bulging, a restrictive right-sided filling pattern, tricuspid E/e′, and hepatic venous flow — had not been systematically recorded, and indeterminate estimates were therefore not adjusted further. An indeterminate pattern was present in 11 of the 30 native-cohort patients (a normal-sized non-collapsing vessel in 6 and a dilated collapsing vessel in 5).

Doppler-estimated right ventricular systolic pressure was the sum of the tricuspid regurgitant pressure gradient and the estimated right atrial pressure. Because invasive hemodynamic measurements were not systematically available at the time of baseline echocardiography, cohort-level values are reported as Doppler-estimated right ventricular systolic pressure rather than as measured pulmonary artery pressures.

An elevated Doppler-estimated right ventricular systolic pressure was defined as a value of 35 mmHg or more. Tricuspid regurgitation severity was taken from the clinical echocardiographic assessment and graded as trivial, mild, moderate, or severe according to current recommendations (13).

#### 2.4.2 Signal-quality grading

The quality of each tricuspid regurgitant signal was graded from 1 to 3 on the completeness of the continuous-wave envelope and the identifiability of its peak, with grade 3 denoting a complete and unambiguous envelope and grade 1 an incomplete envelope whose peak could be identified only with reduced confidence. Velocities were measurable in all recordings, including those graded 1. Beat-to-beat variation in velocity was not used as a grading criterion, because it reflects the underlying physiology of atrioventricular dissociation rather than measurement error. Grading was performed by the same single investigator who made all measurements. An independent second reading of the paired studies is described in Section 2.4.5.

#### 2.4.3 Atrioventricular dissociation

Complete atrioventricular dissociation was confirmed on the simultaneously recorded electrocardiogram and was characterized on Doppler by recurring transmitral A waves and annular a′ waves whose timing varied relative to ventricular diastole. In two patients an A wave following an E wave could not be measured separately because of E–A fusion, which reflects the variable timing of atrial contraction within the cardiac cycle rather than a limitation of image quality.

#### 2.4.4 Aortic valve assessment

Aortic stenosis severity was based on the clinical echocardiographic assessment. Because complete atrioventricular block produces marked beat-to-beat variation in preload and stroke volume per ventricular beat, transvalvular velocities and pressure gradients may be affected by rhythm-related loading conditions. The anatomic valve area obtained by direct planimetry in the parasternal short-axis view was therefore used to define the exclusion threshold. Patients with a planimetered aortic valve area below 1.5 cm^2^ were excluded during cohort selection. All aortic stenosis in the analyzed cohort was mild.

#### 2.4.5 *Post hoc* measurement analyses

Two *post hoc* measurement analyses were performed in the paired cohort in response to peer review; the primary analysis was not changed. First, to assess the influence of beat selection, the primary reader re-measured peak tricuspid regurgitant velocity in the first three consecutive beats with an identifiable peak, counted from the start of the same continuous-wave recording used for the primary measurement. The three velocities were averaged before the pressure gradient was calculated, and the original estimated right atrial pressure was retained, so that only the beat-selection rule differed. Recordings without three consecutive identifiable peaks were treated as missing rather than substituted. This rule was defined before re-measurement.

Second, an independent echocardiographer re-read all 28 studies of the 14 paired patients, measuring peak tricuspid regurgitant velocity by the primary beat-selection rule, grading signal quality, and measuring the inferior vena cava diameter and its inspiratory collapse, from which the estimated right atrial pressure was assigned by the same rule. The second reader was unaware of the primary reader's measurements and the original clinical reports, and the studies were presented in randomized order. Patient identifiers were retained for file handling, and the reader was instructed not to compare the paired study from the same patient. Pacing state could not be concealed because it was evident from the electrocardiographic tracing.

### 2.5 Right heart catheterization

Right heart catheterization was performed through the right femoral vein using a 7-French sheath. A Terumo J-tipped guidewire was positioned in the main pulmonary artery and exchanged for a 7-French Swan-Ganz catheter. Pressures were recorded and blood samples obtained from the right pulmonary artery, main pulmonary artery, right ventricle, right atrium, superior vena cava, and inferior vena cava.

Pressures were recorded at end-expiration with the transducer zeroed at the mid-thoracic level, and values were taken from the automated beat-by-beat measurements provided by the recording system. Because atrioventricular dissociation produces beat-to-beat variation in ventricular filling, a representative beat whose automated values lay closest to the median of the beat-by-beat measurements across the recorded sequence was selected, and its systolic, diastolic, and mean pressures were reported together, so that neither the highest nor the lowest beat determined the reported pressure. The number of beats displayed in each recording was not documented.

Cardiac output was calculated by the indirect Fick method using the same institutional calculation template in all four patients. Oxygen consumption was not measured; a fixed weight-indexed value of 3 mL per kilogram per minute was assumed rather than a published estimating equation such as those of LaFarge or Dehmer. The arteriovenous oxygen content difference was calculated as the difference between systemic arterial and pulmonary arterial oxygen saturation multiplied by the hemoglobin concentration measured on the day of catheterization and an oxygen-carrying capacity of 1.36 mL per gram of hemoglobin; the directly measured pulmonary arterial saturation was used as the mixed venous saturation. Systemic arterial oxygen saturation was obtained by direct arterial sampling in the two sinus-rhythm patients and by pulse oximetry in the two atrial fibrillation comparators, in whom arterial access was not otherwise required. Body surface area was calculated by the Mosteller formula. The transpulmonary gradient was calculated as the mean pulmonary artery pressure minus the pulmonary capillary wedge pressure, and pulmonary vascular resistance as the transpulmonary gradient divided by cardiac output, expressed in Wood units. All procedures were performed and interpreted by a single operator.

Wedge tracings were reviewed for adequacy. In one patient, the tracings obtained in the native state and on day 1 of temporary VVI pacing were damped, with identical systolic, diastolic, and mean values and no discernible a or v waves, indicating over-wedging; in the native state the recorded value also exceeded the mean pulmonary artery pressure. These wedge pressures, and the transpulmonary gradients and resistances derived from them, were treated as not reliable and are not reported.

### 2.6 Derived electrocardiographic variables

The RR and PP intervals on the pre-implantation electrocardiogram were measured manually by the same investigator who performed the echocardiographic measurements, because the interval between successive *P* waves is not reported by the automated analysis when atrial and ventricular activity are dissociated. Ventricular and atrial rates were derived from these intervals. The RR/PP ratio was calculated as the ventricular cycle length divided by the atrial cycle length and therefore expresses the average number of atrial cycles occurring per ventricular cycle. This ratio was evaluated as an exploratory index of the disparity between atrial and ventricular rates; it was not prespecified in the ethics-approved protocol.

All other electrocardiographic intervals were taken from the automated electrocardiographic analysis. QRS duration was obtained in this way from both the pre-implantation electrocardiogram and the post-implantation electrocardiogram used to confirm atrial tracking, so that the paired values were derived by the same algorithm, and the change in QRS duration was calculated as the paced value minus the baseline value in the same patient. The QTc and *P*-to-paced-QRS intervals were obtained in the same way.

### 2.7 Statistical analysis

Continuous variables are summarized as mean ± standard deviation where the distribution was approximately symmetric and as median (interquartile range) otherwise, and categorical variables as counts with percentages. Paired continuous variables were compared with the Wilcoxon signed-rank test and paired binary variables with the exact McNemar test, using only patients in whom both measurements were available. Correlations were assessed with the Spearman rank correlation coefficient. No adjustment for multiple comparisons was applied to secondary or exploratory analyses; these findings were interpreted as supportive or hypothesis-generating, and correlation coefficients were not compared formally with one another. Missing values were not imputed; each analysis used all patients with the relevant measurement available, and the number analyzed is reported for every variable.

Three *post hoc* sensitivity analyses were performed: exclusion of patients with any recorded aortic stenosis (all of which was mild); exclusion of patients with chronic lung disease, defined as a recorded diagnosis of asthma, chronic obstructive pulmonary disease, emphysema, or bronchiectasis; and restriction of the paired comparison to patients in whom the tricuspid regurgitant signal-quality grade did not fall after pacing, that is, in whom it was unchanged or improved. The results of all three are reported in the Supplementary Material.

Further *post hoc* analyses were performed in response to peer review. Baseline characteristics of patients with and without paired studies were compared with the Mann–Whitney U test or Fisher's exact test and with standardized mean differences, calculated after logarithmic transformation for NT-proBNP. Inter-reader agreement for peak tricuspid regurgitant velocity was assessed separately in the native and paced states with the two-way random-effects, absolute-agreement, single-measure intraclass correlation coefficient and by Bland–Altman analysis, with bias calculated as the second-reader value minus the primary-reader value. Agreement for signal-quality grade and estimated right atrial pressure category was assessed with linearly weighted Cohen's *κ*, with 95% confidence intervals from a patient-level cluster bootstrap, and with exact agreement; inferior vena cava diameter was assessed with the same intraclass correlation coefficient across all 28 studies. Paired comparisons in these analyses used the exact Wilcoxon signed-rank test. Treatments given between the paired studies were ascertained from the medical records and summarized descriptively without statistical testing. These analyses are reported in Supplementary Tables S3–S5.

A two-sided *P* value below 0.05 was considered to indicate statistical significance. Analyses were performed in Python 3.12.3 using pandas 3.0.2, NumPy 2.4.4, and SciPy 1.17.1, and figures were produced with Matplotlib 3.10.8. All calculations were carried out on unrounded values; conventional round-half-away-from-zero rounding was applied only for tabular presentation. As a computational reproducibility check, all reported analyses were separately recalculated from the source data using separately generated code in a second environment (Python 3.13.5, NumPy 2.3.5, SciPy 1.17.0); the recalculated results were concordant with the reported results. This procedure was regarded as a separate computational cross-check rather than as blinded or independent validation. The analysis code used in the primary environment was generated with the assistance of Claude (Anthropic, San Francisco, CA, USA; Claude Opus 5), and the code used for the second recalculation was generated separately with the assistance of ChatGPT (OpenAI, San Francisco, CA, USA; GPT-5.6 Sol). Code for the analyses added during revision was generated with the assistance of Claude (Claude Opus 5.5). All analyses were run on the original study data, and all numerical outputs were checked against the source data by the authors.

## 3 Results

### 3.1 Study population and baseline characteristics

Of 142 consecutive permanent pacemaker implantations performed between January 2021 and May 2026, 59 were performed for complete atrioventricular block. Twenty-four patients were excluded for rhythm, imaging, structural, or device-related reasons, leaving 35 eligible patients. Five of these were already receiving temporary VVI pacing at the time of the baseline echocardiogram and were excluded from quantitative analyses because no unpaced reference study was available, giving a native cohort of 30 patients. Fourteen underwent repeat echocardiography within 72 h of atrioventricular-synchronous pacing and formed the primary paired cohort, and two underwent serial echocardiography across pacing states (Figure 1).

**Figure 1:**
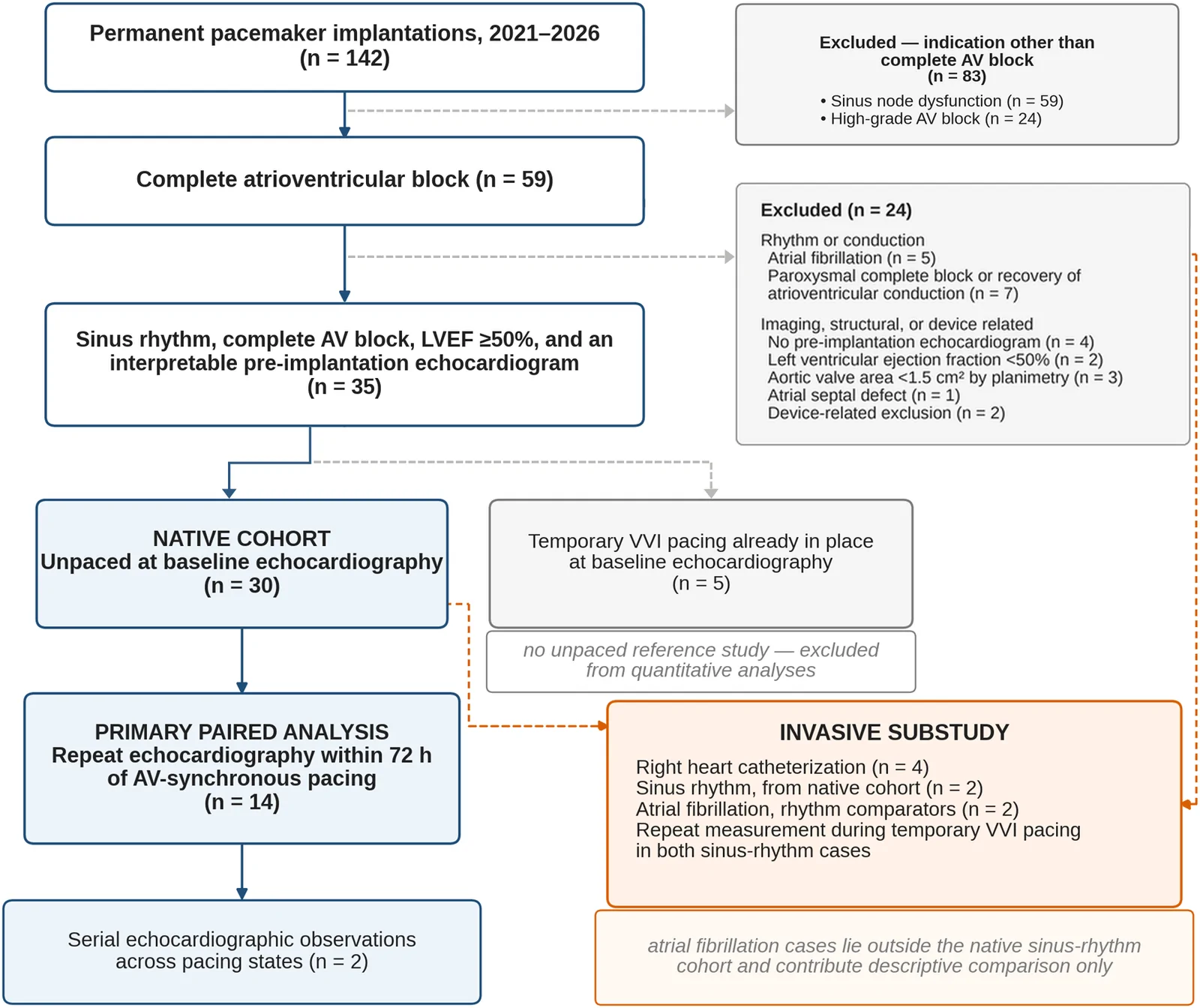
Study flow of 142 consecutive permanent pacemaker implantations performed between January 2021 and May 2026, 59 were performed for complete atrioventricular block. Twenty-four of these were excluded for the rhythm, imaging, structural, or device-related reasons shown, leaving 35 patients in sinus rhythm with complete atrioventricular block, a left ventricular ejection fraction of at least 50%, and an interpretable pre-implantation echocardiogram. Five patients were already receiving temporary VVI pacing at the time of that echocardiogram and were excluded from quantitative analyses because no unpaced reference study was available, giving a native cohort of 30 patients. Fourteen of these underwent repeat echocardiography within 72 h of atrioventricular-synchronous pacing and formed the primary paired cohort, and two underwent serial echocardiography across native rhythm, temporary VVI pacing, and atrioventricular-synchronous pacing. Four patients underwent right heart catheterization on clinical grounds (orange): two were in sinus rhythm and belonged to the native cohort, and two had atrial fibrillation with complete atrioventricular block and lie outside the native sinus-rhythm cohort, contributing descriptive comparison only. Both sinus-rhythm cases underwent repeat invasive measurement during temporary VVI pacing. AV, atrioventricular; LVEF, left ventricular ejection fraction; VVI, ventricular-inhibited pacing.

The mean age was 76.0 ± 11.9 years and 15 patients (50.0%) were men. Dyspnea was the most common presenting symptom (18 patients, 60.0%), followed by dizziness (5, 16.7%). Hypertension was present in 23 patients (76.7%), diabetes mellitus in 9 (30.0%), chronic lung disease in 5 (16.7%), and mild aortic stenosis in 6 (20.0%). The median ventricular rate on the baseline electrocardiogram was 38 beats/min [interquartile range (IQR), 33–44] and the median atrial rate was 83 beats/min (IQR, 72–94), corresponding to a median RR/PP ratio of 2.19 (IQR, 1.95–2.40). The QRS duration was 120 ms or greater in 16 patients (53.3%). Twenty-seven patients received a transvenous dual-chamber system, two a leadless dual-chamber system (Aveir DR), and one a single-component leadless device (Micra AV); the pacing mode was DDD in 29 patients and VDD in 1. The median post-implantation *P*-to-paced-QRS interval was 182 ms (IQR, 175–199). The paced QRS duration was wider than the baseline escape QRS, increasing from a median of 129 to 170 ms (median change, +49 ms; *P* < 0.001), and was wider in 28 of the 30 patients (Table 1).

**Table 1:** Clinical, electrocardiographic, and pacemaker characteristics of the native sinus-rhythm cohort (*n* = 30).

Characteristic	Value
Age, years	76.0 ± 11.9
Male sex	15 (50.0)
Body surface area, m^2^	1.66 ± 0.17
Primary presenting symptom	
Dyspnea	18 (60.0)
Dizziness	5 (16.7)
Syncope or transient loss of consciousness	1 (3.3)
Other	6 (20.0)
Comorbidity	
Hypertension	23 (76.7)
Diabetes mellitus	9 (30.0)
Coronary artery disease	6 (20.0)
Previous stroke	7 (23.3)
Chronic lung disease	5 (16.7)
Mild aortic stenosis	6 (20.0)
Laboratory	
Creatinine, mg/dL	0.99 (0.75–1.19)
eGFR, mL/min/1.73 m^2^	70.3 ± 22.2
Hemoglobin, g/dL	12.5 ± 2.1
NT-proBNP, pg/mL (*n* = 28)	1,850 (520–3,410)
Baseline electrocardiogram	
Ventricular rate, beats/min	38 (33–44)
RR interval, ms	1,600 (1,372–1,805)
Atrial rate, beats/min	83 (72–94)
PP interval, ms	720 (640–830)
RR/PP ratio	2.19 (1.95–2.40)
QRS duration, ms	129 (95–140)
QRS duration ≥120 ms	16 (53.3)
QTc interval, ms	463 (442–517)
Pacemaker system	
Transvenous system	27 (90.0)
Leadless system	3 (10.0)
DDD mode	29 (96.7)
VDD mode	1 (3.3)
*P*-to-paced-QRS interval, ms	182 (175–199)
Paced QRS duration, ms	170 (155–188)
Change in QRS duration, ms	+49 (+32 to +74)

Values are mean ± standard deviation, median (interquartile range), or *n* (%). Categories of primary presenting symptom are mutually exclusive. Other primary presenting symptoms were chest pain, chest discomfort, vomiting, general weakness, edema, and headache (one patient each). The RR/PP ratio is the ventricular cycle length divided by the atrial cycle length, and therefore expresses the average number of atrial cycles occurring per ventricular cycle. The *P*-to-paced-QRS interval was measured from the surface electrocardiogram at the post-implantation study and is reported as a marker of atrial tracking; programmed atrioventricular delay values were not recorded. Paced QRS duration was measured on the same post-implantation electrocardiogram, and the change in QRS duration is the paced value minus the baseline value in the same patient. Leadless systems comprised two Aveir DR dual-chamber systems (communicating right atrial and right ventricular components; DDD) and one Micra AV (VDD). All leadless ventricular components were mid-septal; of 27 transvenous ventricular leads, 23 were apical and 4 in the left bundle branch area. eGFR, estimated glomerular filtration rate; NT-proBNP, N-terminal pro-B-type natriuretic peptide.

### 3.2 Echocardiographic phenotype during native complete atrioventricular block

At the baseline study the median heart rate was 41 beats/min (IQR, 37–47). The peak tricuspid regurgitant velocity was 3.03 m/s (IQR, 2.52–3.28), corresponding to a tricuspid regurgitant pressure gradient of 36.7 mmHg (IQR, 25.4–42.9). The estimated right atrial pressure was 8 mmHg (IQR, 3–8); an indeterminate inferior vena cava pattern was present in 11 of the 30 patients. The median Doppler-estimated right ventricular systolic pressure (RVSP) was 44.0 mmHg (IQR, 32.4–50.3). Doppler-estimated RVSP was 35 mmHg or greater in 21 patients (70.0%) and 40 mmHg or greater in 20 (66.7%), and the peak tricuspid regurgitant velocity was 2.9 m/s or greater in 18 patients (60.0%) (Table 2).

**Table 2:** Echocardiographic phenotype during native complete atrioventricular block (n = 30).

Variable	Value
Heart rate during echocardiography, beats/min	41 (37–47)
Right-sided hemodynamics	
Peak tricuspid regurgitant velocity, m/s	3.03 (2.52–3.28)
Peak tricuspid regurgitant pressure gradient, mmHg	36.7 (25.4–42.9)
Estimated right atrial pressure, mmHg	8 (3–8)
Doppler-estimated RVSP, mmHg	44.0 (32.4–50.3)
Elevated Doppler-estimated RVSP (≥35 mmHg)	21 (70.0)
Doppler-estimated RVSP ≥40 mmHg	20 (66.7)
Peak tricuspid regurgitant velocity ≥2.9 m/s	18 (60.0)
Inferior vena cava diameter, cm	1.84 (1.61–2.17)
RV/LV basal diameter ratio	0.86 (0.81–0.91)
Tricuspid regurgitation	
Grade 0 (trivial)	13 (43.3)
Grade 1 (mild)	15 (50.0)
Grade 2 (moderate)	2 (6.7)
Tricuspid regurgitant signal quality	
Grade 1	5 (16.7)
Grade 2	8 (26.7)
Grade 3	17 (56.7)
Left heart	
Left ventricular ejection fraction, %	64 (62–66)
LV end-diastolic diameter, mm	50.0 (47.3–53.8)
LV outflow tract velocity–time integral, cm (*n* = 19)	30.2 (25.7–33.9)
LV stroke volume, mL (*n* = 19)	100.7 (90.8–114.7)
LV cardiac output, L/min (*n* = 19)	4.24 (3.03–4.73)
E/e′	16.5 (12.9–19.6)
Left atrial volume index, mL/m^2^ (*n* = 27)	50.3 (39.2–55.5)
Atrioventricular dissociation	
Complete atrioventricular dissociation	30 (100.0)
Transmitral A-wave velocity following an E wave, m/s (*n* = 28)	1.01 (0.75–1.17)
Dissociated A-wave velocity, m/s (*n* = 30)	0.75 (0.56–0.98)
Dissociated a′ velocity, cm/s (*n* = 30)	6.0 (5.0–7.2)

Values are median (interquartile range) or *n* (%). Right ventricular systolic pressure was estimated as the peak tricuspid regurgitant pressure gradient plus the right atrial pressure estimated from inferior vena cava diameter and respiratory collapse, assigned as 3, 8, or 15 mmHg in accordance with current American Society of Echocardiography recommendations; an indeterminate pattern was present in 11 patients. Because invasive hemodynamic measurements were not systematically available at the time of baseline echocardiography and were obtained in only two patients of this cohort, cohort-level values are reported as Doppler-estimated RVSP rather than as measured pulmonary artery pressures. Peak tricuspid regurgitant velocity was measured from continuous-wave recordings obtained from multiple acoustic windows. Because ventricular filling varies from beat to beat when atrial and ventricular activity are dissociated, beats were first screened for the most complete envelope with an identifiable peak within each recording, and the highest velocity among those beats was taken; the same rule was applied to every recording regardless of its overall signal quality. Tricuspid regurgitant signal quality was graded on the completeness of the continuous-wave envelope and the identifiability of its peak, grade 3 denoting a complete and unambiguous envelope; beat-to-beat velocity variation was not used as a grading criterion because it reflects physiology rather than measurement error. Complete atrioventricular dissociation was confirmed on the simultaneously recorded electrocardiogram and was characterized on Doppler by recurring transmitral A waves and annular a′ waves whose timing varied relative to ventricular diastole. Dissociated A-wave and a′ velocities were measurable in all 30 patients. In two patients an A wave following an E wave could not be measured separately because of E–A fusion, which reflects the variable timing of atrial contraction within the cardiac cycle rather than a limitation of image quality. AV, atrioventricular; LV, left ventricular; RV, right ventricular; RVSP, right ventricular systolic pressure.

Tricuspid regurgitation was trivial in 13 patients (43.3%), mild in 15 (50.0%), and moderate in 2 (6.7%); no patient had severe regurgitation. The tricuspid regurgitant signal was graded 3 in 17 patients (56.7%), grade 2 in 8 (26.7%), and grade 1 in 5 (16.7%), and a peak velocity was measurable in every recording. The right-to-left ventricular basal diameter ratio was 0.86 (IQR, 0.81–0.91).

Left ventricular systolic function was preserved, with a median ejection fraction of 64% (IQR, 62–66). Among the 19 patients with interpretable outflow-tract flow measurements, the stroke volume was 100.7 mL (IQR, 90.8–114.7) and the cardiac output was 4.24 L/min (IQR, 3.03–4.73). Complete atrioventricular dissociation was present in all 30 patients. Dissociated transmitral A waves and annular a′ waves were measurable in every patient, whereas an A wave following an E wave could not be measured separately in two patients because of E–A fusion (Table 2; Supplementary Figure S1).

### 3.3 Changes after atrioventricular-synchronous pacing

The paired echocardiograms were separated by a median of 2 days (range, 1–8), and every post-implantation study was obtained within 72 h of the procedure. Atrioventricular-synchronous pacing raised the median heart rate from 38 to 67 beats/min (*P* < 0.001).

The primary endpoint, Doppler-estimated RVSP, fell from 46.6 mmHg (IQR, 37.5–59.2) during native complete atrioventricular block to 25.0 mmHg (IQR, 15.5–29.3) after atrioventricular-synchronous pacing, a median change of −18.0 mmHg (*P* < 0.001). Doppler-estimated RVSP decreased in all 14 patients, and the median patient-level relative change was −51.4% (IQR, −57.7% to −34.2%). The proportion with a value of 35 mmHg or greater fell from 11 of 14 (78.6%) to 3 of 14 (21.4%; exact McNemar *P* = 0.008) (Table 3; Figure 2A).

**Table 3:** Paired echocardiographic measurements before and within 72 h after atrioventricular-synchronous pacing (*n* = 14).

Variable	n	Native complete AV block	AV-synchronous pacing	Median change	*P* value
Heart rate, beats/min	14	38 (33–44)	67 (63–76)	+33	<0.001
Peak tricuspid regurgitant velocity, m/s	14	3.06 (2.58–3.48)	2.34 (1.77–2.57)	−0.67	0.001
Peak tricuspid regurgitant pressure gradient, mmHg	14	37.5 (26.6–48.5)	22.0 (12.5–26.3)	−14.5	0.001
Inferior vena cava diameter, cm	14	2.01 (1.80–2.22)	1.59 (1.37–1.78)	−0.27	0.019
Estimated right atrial pressure, mmHg	14	8 (8–15)	3 (3–3)	−5	0.003
Doppler-estimated RVSP, mmHg	14	46.6 (37.5–59.2)	25.0 (15.5–29.3)	−18.0	<0.001
Left ventricular ejection fraction, %	14	64.0 (62.5–65.0)	62.5 (61.0–64.8)	−1	0.144
LV outflow tract velocity–time integral, cm*	7	31.8 (28.7–34.8)	20.7 (18.6–24.6)	−11.0	0.016
LV stroke volume, mL*	7	104.0 (93.6–113.6)	69.0 (61.0–73.9)	−40.5	0.016
LV cardiac output, L/min*	7	3.34 (2.87–4.64)	4.56 (4.20–4.67)	+0.59	0.078
E/e′*	9	13.5 (10.5–21.3)	11.6 (9.4–12.1)	−1.4	0.008
Tricuspid regurgitation grade	14	1 (0–1)	0 (0–0)	−0.5	0.008
Tricuspid regurgitant signal quality grade	14	3 (2–3)	2 (1–2)	−0.5	0.070
Elevated Doppler-estimated RVSP (≥35 mmHg), *n* (%)	14	11 (78.6)	3 (21.4)	—	0.008
Peak tricuspid regurgitant velocity ≥2.9 m/s, *n* (%)	14	9 (64.3)	2 (14.3)	—	0.016

Values are median (interquartile range) or *n* (%). Changes were calculated as the post-pacing value minus the native complete-AV-block value. *P* values are from the Wilcoxon signed-rank test, except for the two dichotomous rows, which were compared using the exact McNemar test.

*Exploratory analyses: paired outflow-tract flow measurements were available in 7 patients and paired E/e′ in 9. The median patient-level relative change in Doppler-estimated RVSP was −51.4% (interquartile range −57.7 to −34.2%); Doppler-estimated RVSP decreased in all 14 patients. Although the interval between the paired echocardiograms ranged from 1 to 8 days (median 2, interquartile range 1–3), every post-implantation study was obtained within 72 h of the procedure. The change in heart rate was not correlated with the change in Doppler-estimated RVSP (Spearman *ρ* = −0.145, *P* = 0.620); with 14 paired observations this analysis has limited power and does not exclude a rate-related contribution. Tricuspid regurgitant signal quality also tended to decline after pacing, so the fall in Doppler-estimated RVSP should be interpreted alongside the change in envelope quality. In the native state the highest-velocity beat with an adequate envelope was selected. After atrioventricular-synchronous pacing the Doppler profiles were substantially more uniform and little beat selection was generally required. Because beat-to-beat variability was greater in the native state, this approach may have accentuated the magnitude of the reduction in peak Doppler estimates. In a *post hoc* sensitivity analysis using the mean of three consecutive beats, the reduction was of similar magnitude in the six patients with measurable pairs (Supplementary Table S4). Abbreviations as in Table 2.

**Figure 2:**
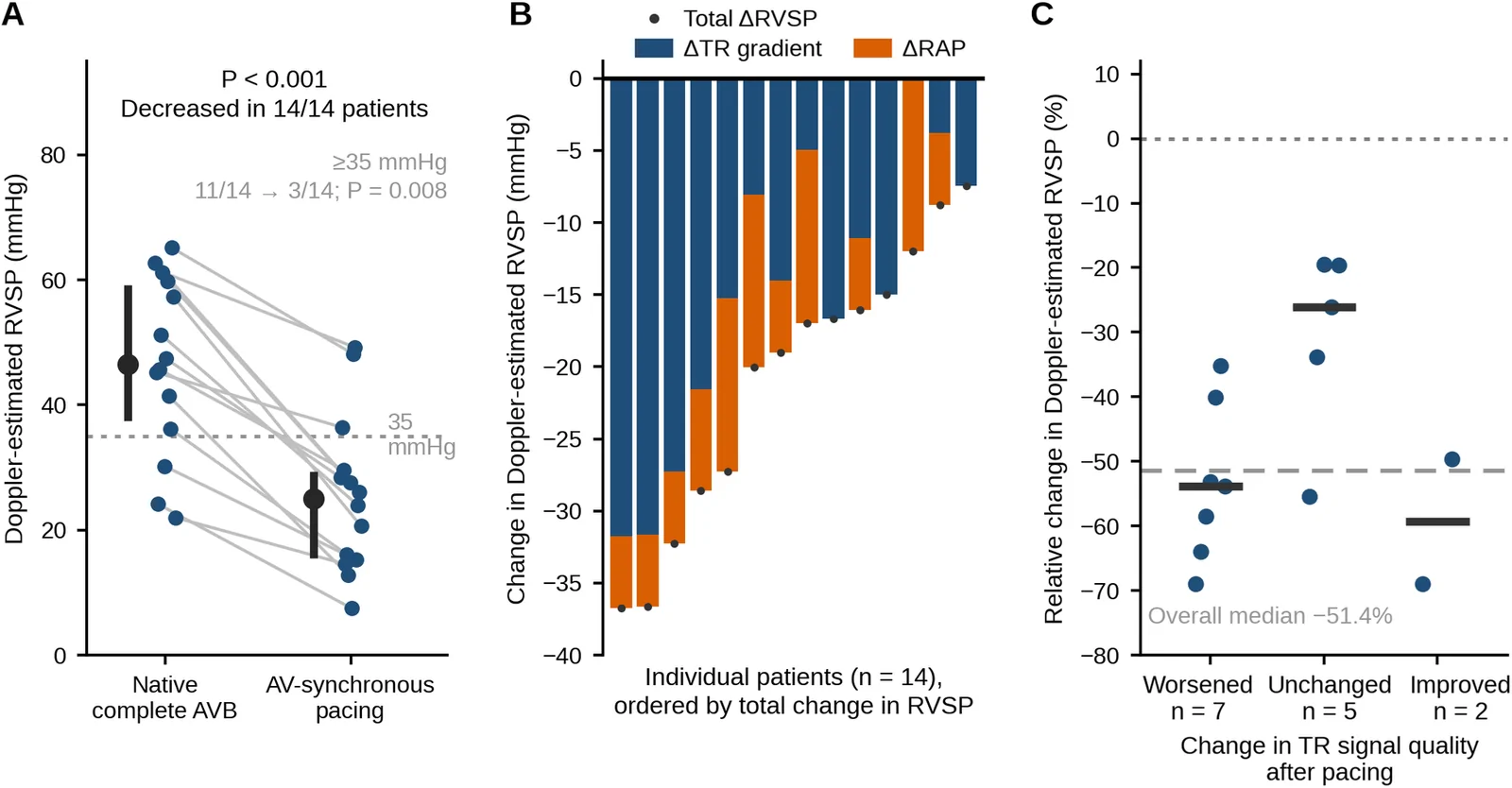
Change in Doppler-estimated right ventricular systolic pressure after atrioventricular-synchronous pacing in the paired cohort (*n* = 14). **(A)** Individual paired values during native complete atrioventricular block and after atrioventricular-synchronous pacing; gray lines connect the two studies in the same patient and black symbols show the median with the interquartile range. Doppler-estimated right ventricular systolic pressure fell in every patient (Wilcoxon signed-rank test), and the proportion with an elevated value fell from 11 of 14 to 3 of 14 (exact McNemar test). The dotted line marks the 35 mmHg threshold used to define an elevated value. **(B)** Patient-level change in Doppler-estimated right ventricular systolic pressure partitioned into its two components, the change in tricuspid regurgitant pressure gradient (blue) and the change in estimated right atrial pressure (orange), with patients ordered by total change; black dots mark the total change, which ranged from −36.7 to −7.4 mmHg. In one patient the peak tricuspid regurgitant velocity was identical before and after pacing, so the entire change in that patient arose from the estimated right atrial pressure component. **(C)** Relative change in Doppler-estimated right ventricular systolic pressure stratified by the change in tricuspid regurgitant signal-quality grade after pacing; horizontal bars are stratum medians (worsened −53.9%, unchanged −26.1%, improved −59.3%), the dashed line is the overall median of −51.4%, and the dotted line marks no change. Because signal quality declined in half of the cohort, the fall in Doppler-estimated right ventricular systolic pressure should be interpreted alongside the change in envelope quality. RAP, right atrial pressure; RVSP, right ventricular systolic pressure; TR, tricuspid regurgitant.

Both components of the estimate decreased. The peak tricuspid regurgitant velocity fell from 3.06 to 2.34 m/s (*P* = 0.001) and the corresponding pressure gradient from 37.5 to 22.0 mmHg (*P* = 0.001), while the inferior vena cava diameter decreased from 2.01 to 1.59 cm (*P* = 0.019) and the estimated right atrial pressure from 8 to 3 mmHg (*P* = 0.003). The relative contribution of the two components varied between patients (Figure 2B). The proportion with a peak tricuspid regurgitant velocity of 2.9 m/s or greater fell from 9 of 14 (64.3%) to 2 of 14 (14.3%; *P* = 0.016).

Tricuspid regurgitation grade also fell, decreasing in 7 patients and increasing in none (*P* = 0.008); after pacing, regurgitation was trivial in 11 patients and mild in 3. Tricuspid regurgitant signal quality tended to decline over the same interval, from a median grade of 3 to 2 (*P* = 0.070), deteriorating in 7 patients, remaining unchanged in 5, and improving in 2 (Figure 2C). The change in heart rate was not correlated with the change in Doppler-estimated RVSP (Spearman *ρ* = −0.145, *P* = 0.620).

In the exploratory paired measurements, the left ventricular outflow-tract velocity–time integral fell from 31.8 to 20.7 cm and the stroke volume from 104.0 to 69.0 mL (both *P* = 0.016, *n* = 7), whereas the cardiac output rose slightly, from 3.34 to 4.56 L/min, without reaching statistical significance (*P* = 0.078). E/e′ fell from 13.5 to 11.6 (*P* = 0.008, *n* = 9). The left ventricular ejection fraction was unchanged (*P* = 0.144) (Table 3).

### 3.4 Invasive hemodynamic observations

Right heart catheterization was performed in four patients: two in sinus rhythm who belonged to the native cohort, and two with atrial fibrillation and complete atrioventricular block who lie outside that cohort and are reported as descriptive rhythm comparators (Table 4). The invasive mean pulmonary artery pressure was 31 and 38 mmHg in the two sinus-rhythm cases and 18 and 19 mmHg in the two atrial fibrillation comparators, whose ventricular rates were 37 and 55 beats/min. No formal comparison between rhythm groups was performed.

**Table 4:** Invasive hemodynamic measurements in four patients with complete atrioventricular block.

Case	Age/sex	Rhythm	State	HR	sPAP	mPAP	PCWP	TPG	PVR	CO
Case 1	71/M	AF	Native	37	28	18	13	+5	0.82	6.11
Case 2	84/F	AF	Native	55	27	19	18	+1	0.15	6.77
Case 3^‡^	72/F	SR	Native	46	52	31	17	+14	3.51	3.98
			Temporary VVI pacing, day 1	60	45	29	13	+16	3.44	4.65
Case 4^†^	70/M	SR	Native	29	60	38	NR^†^	—	NR^†^	3.93
			Temporary VVI pacing, day 1	60	52	43	NR^†^	—	NR^†^	—
			Temporary VVI pacing, day 8	60	31	28	23	+5	1.10	4.53

Pressures are in mmHg, cardiac output in L/min and heart rate in beats/min. Heart rate during temporary VVI pacing is the programmed pacing rate. Pulmonary vascular resistance is expressed in Wood units and calculated as the transpulmonary gradient divided by cardiac output. Invasive pressures were recorded at end-expiration with the transducer zeroed at the mid-thoracic level, and were taken from a representative beat closest to the median of the automated beat-by-beat values. In Case 2 the transpulmonary gradient was 1 mmHg, so the derived pulmonary vascular resistance is at the limit of measurement precision and should not be interpreted quantitatively.

^†^In Case 4 the wedge tracings in the native state and on day 1 of temporary VVI pacing were damped, with identical systolic, diastolic and mean values and no discernible a or v waves, indicating over-wedging; in the native state the recorded value also exceeded the mean pulmonary artery pressure. These wedge pressures, and the transpulmonary gradients and resistances derived from them, are therefore reported as not reliable (NR). The pulmonary arterial, right ventricular and right atrial tracings from the same studies showed normal phasic contours, left ventricular pressure recorded during the native study was 191/25 mmHg, and the day 8 wedge tracing was of normal contour. Systemic arterial pressure displayed on the monitor during the day 1 study was 253/106 mmHg (Supplementary Figure S2). Cardiac output was not measured separately on day 1 of VVI pacing in this case and is therefore not reported.

^‡^Case 3 had clinically significant bronchiectasis with recurrent hemoptysis; pulmonary vascular resistance was essentially unchanged during VVI pacing while the wedge pressure fell, which may reflect a concomitant pulmonary vascular component. Cases 1 and 2 had atrial fibrillation with complete atrioventricular block and served as descriptive rhythm comparators; they were outside the native sinus-rhythm cohort and did not undergo repeat catheterization. Day 8 measurements are available for Case 4 only. AF, atrial fibrillation; CO, cardiac output; HR, heart rate; mPAP, mean pulmonary artery pressure; NR, not reliable; PCWP, pulmonary capillary wedge pressure; PVR, pulmonary vascular resistance; sPAP, systolic pulmonary artery pressure; SR, sinus rhythm; TPG, transpulmonary gradient; VVI, ventricular-inhibited pacing.

Doppler estimates obtained in the same pacing state diverged from the invasive measurements in both directions (Figure 3A). The Doppler estimate exceeded the invasive systolic pulmonary artery pressure by 57.6 and 18.5 mmHg in the two atrial fibrillation comparators and by 10.8 mmHg in one sinus-rhythm case, but fell 2.7 mmHg below it in the other. Across the four cases no simple relation between ventricular rate and invasive mean pulmonary artery pressure was apparent (Figure 3B).

**Figure 3:**
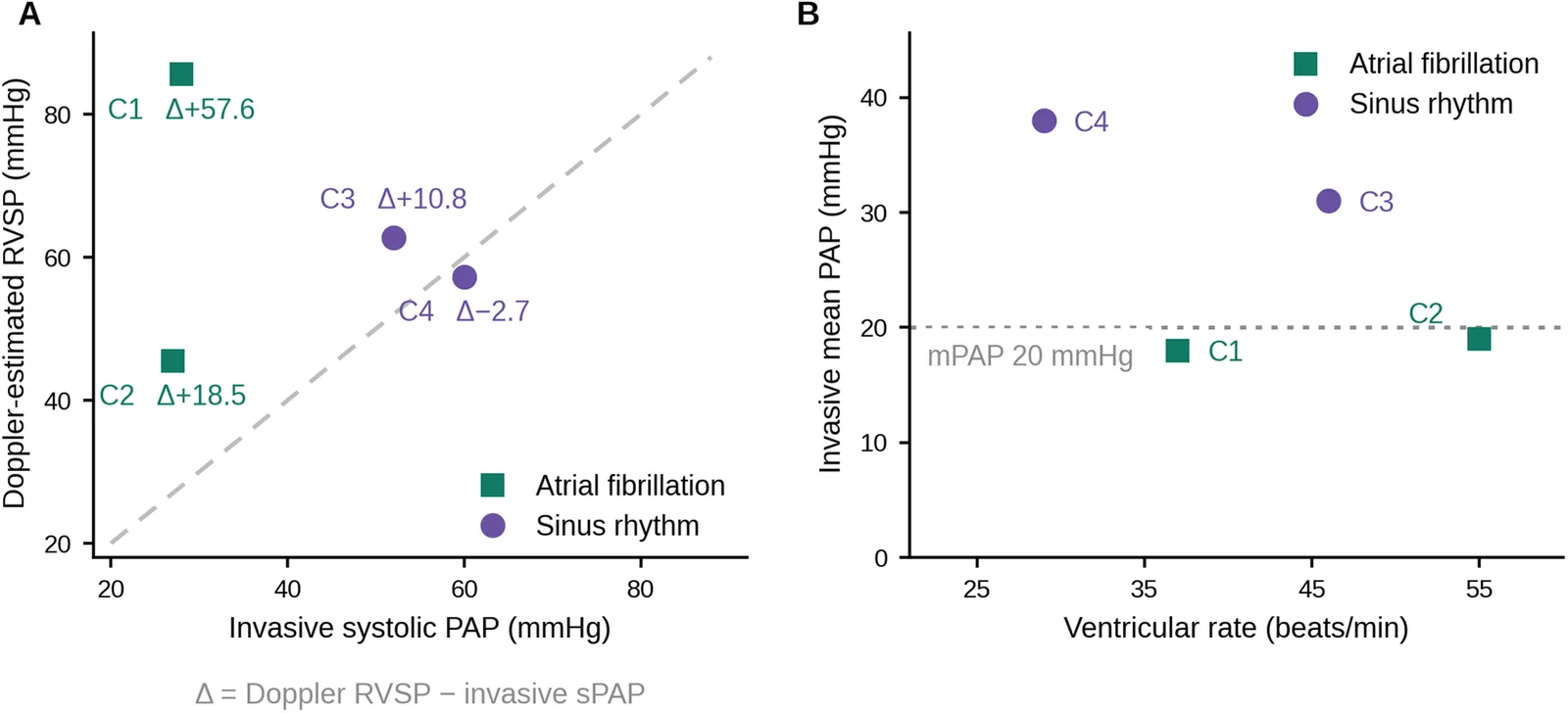
Relation between Doppler-estimated and invasively measured pulmonary artery pressure in the four patients who underwent right heart catheterization. Cases 1 and 2 had atrial fibrillation with complete atrioventricular block (squares) and are descriptive rhythm comparators outside the native sinus-rhythm cohort; Cases 3 and 4 were in sinus rhythm and belonged to the native cohort (circles). **(A)** Doppler-estimated right ventricular systolic pressure plotted against invasive systolic pulmonary artery pressure obtained in the same pacing state; the dashed line is the line of identity, and *Δ* is the Doppler estimate minus the invasive value. Discordance was bidirectional: the Doppler estimate exceeded the invasive value by 57.6 and 18.5 mmHg in the two atrial fibrillation cases and by 10.8 mmHg in one sinus-rhythm case, but fell 2.7 mmHg below it in the other. **(B)** Invasive mean pulmonary artery pressure plotted against ventricular rate at catheterization; the dotted line marks 20 mmHg. Mean pulmonary artery pressure was elevated in both sinus-rhythm cases (ventricular rates 46 and 29 beats/min) but not in either atrial fibrillation comparator (37 and 55 beats/min). No simple relation between ventricular rate and invasive mean pulmonary artery pressure was apparent across these four cases. Doppler estimates in **(A)** were taken from the highest-velocity beat with an adequate envelope, whereas invasive pressures were taken from a representative beat closest to the median of the automated beat-by-beat values. PAP, pulmonary artery pressure; RVSP, right ventricular systolic pressure.

In Case 3, temporary VVI pacing raised the ventricular rate from 46 to 60 beats/min and lowered the pulmonary capillary wedge pressure from 17 to 13 mmHg and the mean pulmonary artery pressure from 31 to 29 mmHg, while the pulmonary vascular resistance remained essentially unchanged (3.51 to 3.44 Wood units) (Figure 4A). In Case 4, the invasive systolic pulmonary artery pressure was 60 mmHg in the native state, 52 mmHg on day 1 of temporary VVI pacing, and 31 mmHg on day 8. The wedge tracings obtained in the native state and on day 1 in this patient were damped and were treated as unreliable, so no transpulmonary gradient or pulmonary vascular resistance is reported for those measurements.

**Figure 4:**
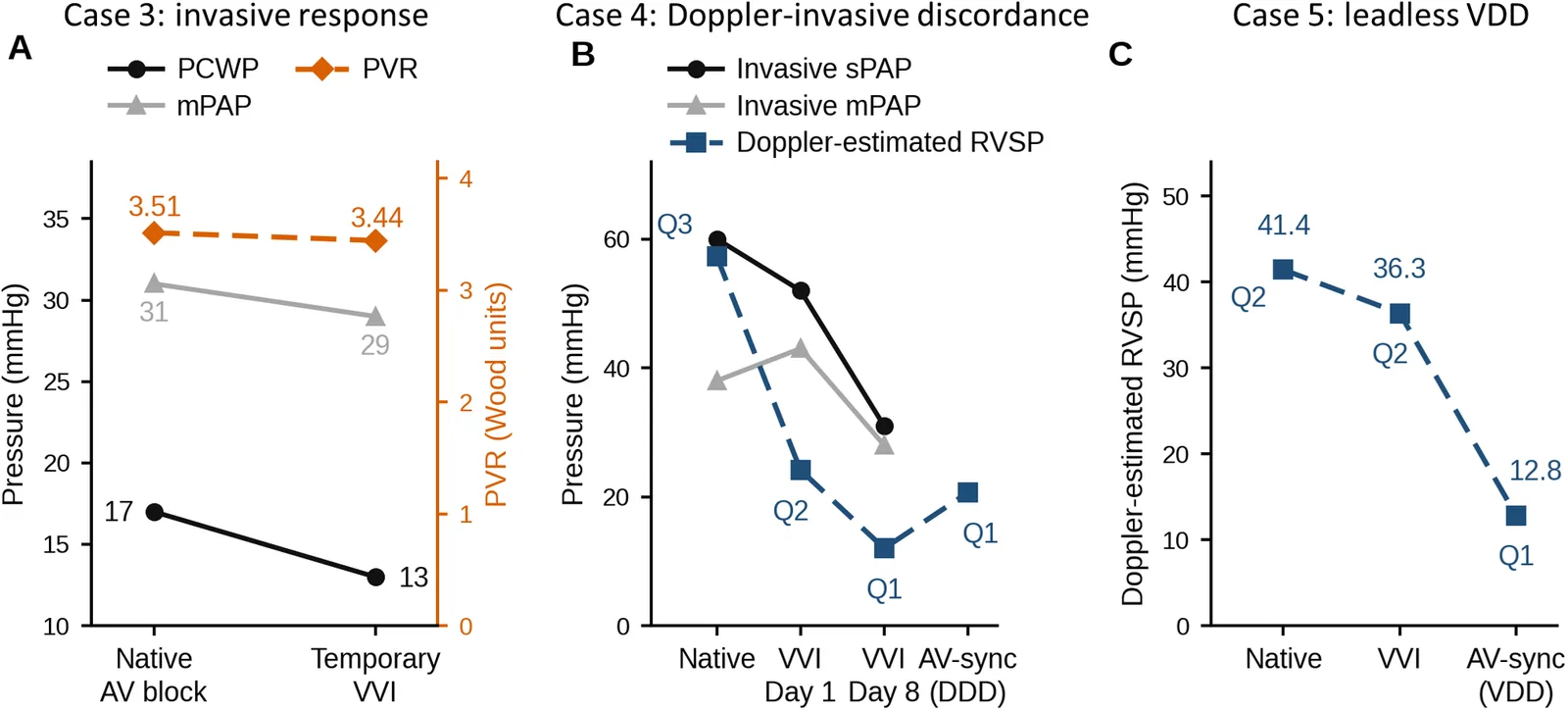
Case-level hemodynamic observations across pacing states. **(A)** Case 3, a sinus-rhythm patient in the native cohort with bronchiectasis: invasive measurements during native complete atrioventricular block and during temporary VVI pacing. Correcting the ventricular rate from 46 to 60 beats/min lowered the pulmonary capillary wedge pressure from 17 to 13 mmHg and the mean pulmonary artery pressure from 31 to 29 mmHg, while pulmonary vascular resistance remained essentially unchanged (3.51 to 3.44 Wood units, right axis). **(B)** Case 4, the same patient as Case 4 in Table 4: invasive systolic and mean pulmonary artery pressure and the same-day Doppler-estimated right ventricular systolic pressure across native complete atrioventricular block, temporary VVI pacing on days 1 and 8, and atrioventricular-synchronous pacing. The Doppler estimate tracked the invasive values in the native state but underestimated them markedly during VVI pacing on day 1 (24.2 versus an invasive systolic pressure of 52 mmHg), coinciding with a fall in tricuspid regurgitant signal quality from grade 3 to grade 2. No invasive measurement was obtained during atrioventricular-synchronous pacing. **(C)** Case 5, a leadless VDD recipient: Doppler-estimated right ventricular systolic pressure during native complete atrioventricular block, temporary VVI pacing, and atrioventricular-synchronous pacing. Ventricular rate and outflow-tract-derived cardiac output were closely similar during VVI pacing (66 beats/min, 4.80 L/min) and atrioventricular-synchronous pacing (67 beats/min, 4.62 L/min), while the Doppler estimate was markedly lower in the latter state. However, intravenous loop diuretic was given before the VVI study and the oral diuretic dose was reduced after it, and the tricuspid envelope during atrioventricular-synchronous pacing was of grade 1 quality, as it was in Case 4. This comparison is therefore descriptive and does not isolate the effect of atrioventricular synchrony or establish a further fall in pulmonary artery pressure. Q1 to Q3 denote the tricuspid regurgitant signal-quality grade. mPAP, mean pulmonary artery pressure; PCWP, pulmonary capillary wedge pressure; PVR, pulmonary vascular resistance; RVSP, right ventricular systolic pressure; sPAP, systolic pulmonary artery pressure; VVI, ventricular-inhibited pacing.

### 3.5 Serial physiologic observations

Two patients underwent serial echocardiography across native complete atrioventricular block, temporary VVI pacing, and atrioventricular-synchronous pacing (Table 5; Figure 4).

**Table 5:** Serial echocardiographic observations across native complete atrioventricular block, temporary VVI pacing, and atrioventricular-synchronous pacing in two illustrative physiologic cases.

Case	State	AV dissociation	HR	TR Vmax	TR gradient	Signal quality	RAP	RVSP	LV CO
Case 4	Native complete AV block	Present	29	3.51	49.3	3	8	57.3	3.34
Case 4	Temporary VVI pacing, day 1	Present	62	2.30	21.2	2	3	24.2	4.37
Case 4	Temporary VVI pacing, day 8	Present	62	1.50	9.0	1	3	12.0	3.30
Case 4	AV-synchronous pacing	Absent	54	2.10	17.6	1	3	20.6	3.94
Case 5	Native complete AV block	Present	43	2.57	26.4	2	15	41.4	4.81
Case 5	Temporary VVI pacing, day 1	Present	66	2.31	21.3	2	15	36.3	4.80
Case 5	AV-synchronous pacing	Absent	67	1.10	4.8	1	8	12.8	4.62

Heart rate is in beats/min, peak tricuspid regurgitant velocity in m/s, pressures and gradients in mmHg, and cardiac output in L/min. Case 4 is the same patient as Case 4 in Table 4; Case 5 did not undergo catheterization. VVI pacing corrects the ventricular rate but does not track atrial activity, so atrioventricular dissociation persists; visible transmitral A waves during VVI pacing represent dissociated atrial contractions that happen to fall within diastole and do not indicate atrioventricular synchrony. In Case 5 the ventricular rate and outflow-tract-derived cardiac output were closely similar during VVI pacing (66 beats/min, 4.80 L/min) and during atrioventricular-synchronous pacing (67 beats/min, 4.62 L/min), yet the tricuspid regurgitant velocity was markedly lower in the latter state. In this patient, intravenous loop diuretic was given between the native and VVI studies, and the oral diuretic dose was reduced between the VVI and atrioventricular-synchronous studies; the estimated right atrial pressure was 15 mmHg in the native and VVI states and 8 mmHg during atrioventricular-synchronous pacing. Loading conditions therefore differed across states, and the comparison remains descriptive. The tricuspid envelope during atrioventricular-synchronous pacing was of grade 1 quality, however, and the lower Doppler estimate should therefore not be read as a demonstrated further fall in pulmonary artery pressure. The same caution applies to the atrioventricular-synchronous state of Case 4. In Case 4 the Doppler estimate obtained during VVI pacing on day 1 was discordant with the contemporaneous invasive measurement reported in Table 4, performed on the same day in the same pacing state. During VVI pacing the ventricular rate is regular but atrial activity remains dissociated, so ventricular filling continues to vary from beat to beat; peak velocities in every state were taken from the highest-velocity beat with an adequate envelope, whereas invasive pressures were taken from a representative beat closest to the median of the automated beat-by-beat values. This asymmetry would, if anything, bias the Doppler estimate upward; beat selection is therefore unlikely to explain the observed underestimation. LV CO, left ventricular cardiac output; RAP, estimated right atrial pressure; RVSP, right ventricular systolic pressure; TR, tricuspid regurgitant.

In Case 4, the Doppler-estimated RVSP was 57.3 mmHg during native complete atrioventricular block, 24.2 mmHg on day 1 of VVI pacing, 12.0 mmHg on day 8, and 20.6 mmHg during atrioventricular-synchronous pacing. The native estimate was close to the same-day invasive systolic pulmonary artery pressure of 60 mmHg, whereas the day-1 estimate of 24.2 mmHg was markedly discordant with the same-day invasive value of 52 mmHg. Over the same sequence the tricuspid regurgitant signal quality fell from grade 3 in the native state to grade 2 on day 1 and grade 1 thereafter (Figure 4B; Supplementary Figure S2).

In Case 5, atrioventricular dissociation persisted during VVI pacing and was absent during atrioventricular-synchronous pacing. The ventricular rate and outflow-tract-derived cardiac output were closely similar in the two paced states (66 vs. 67 beats/min and 4.80 versus 4.62 L/min), while the Doppler-estimated RVSP was 36.3 mmHg during VVI pacing and 12.8 mmHg during atrioventricular-synchronous pacing. Intravenous loop diuretic had been given before the VVI study, and the oral diuretic dose was reduced between the VVI and atrioventricular-synchronous studies, over which the estimated right atrial pressure fell from 15 to 8 mmHg. The tricuspid envelope in the atrioventricular-synchronous state was of grade 1 quality, and this comparison is therefore descriptive rather than evidence of a further fall in pulmonary artery pressure (Figure 4C).

### 3.6 Exploratory associations and sensitivity analyses

In the native cohort, the RR/PP ratio was correlated with the estimated right atrial pressure (*ρ* =  + 0.580, *P* < 0.001) and with Doppler-estimated RVSP (*ρ* =  + 0.428, *P* = 0.018), but not with the tricuspid regurgitant pressure gradient (*ρ* =  + 0.236, *P* = 0.210). Neither the ventricular rate nor the atrial rate showed a statistically significant association with these variables. The coefficients were not compared formally, and all of these analyses were exploratory (Supplementary Table S1).

The paired reduction in Doppler-estimated RVSP persisted in all three *post hoc* sensitivity analyses (Supplementary Table S2). Doppler-estimated RVSP fell from 46.3 to 22.3 mmHg after exclusion of patients with aortic stenosis (*n* = 12; *P* < 0.001) and from 45.5 to 22.3 mmHg after exclusion of patients with chronic lung disease (*n* = 10; *P* = 0.002), with a reduction in every patient in both analyses. Among the 7 patients whose tricuspid regurgitant signal-quality grade did not fall after pacing, Doppler-estimated RVSP fell from 36.2 to 16.1 mmHg (*P* = 0.016) and decreased in all 7; the median relative change in this subgroup was −33.8%, compared with −51.4% in the full paired cohort.

The exploratory association between the RR/PP ratio and Doppler-estimated RVSP was no longer significant after exclusion of patients with chronic lung disease (*ρ* =  + 0.374, *P* = 0.065), whereas its association with the estimated right atrial pressure persisted (*ρ* =  + 0.571, *P* = 0.003).

In the *post hoc* three-beat analysis, three consecutive identifiable beats were available in 9 of 14 native and 10 of 14 paced studies, yielding six complete pairs, in five of which signal quality was unchanged or improved. In these six patients, Doppler-estimated RVSP based on the three-beat mean fell in all six (median relative change −42.7%; *P* = 0.031), compared with −40.8% by the primary method in the same patients, and the difference between the highest-beat velocity and the three-beat mean was not detectably different between the native and paced states (median 0.18 versus 0.12 m/s; *P* = 0.56) (Supplementary Table S4).

Inter-reader agreement for peak tricuspid regurgitant velocity was high in the native state (intraclass correlation coefficient 0.98; bias −0.03 m/s) and lower in the paced state (0.83; bias −0.24 m/s), in which the second reader tended to record lower velocities. Weighted *κ* was 0.84 for signal-quality grade (exact agreement in 24 of 28 studies) and 0.87 for the estimated right atrial pressure category (25 of 28), and the intraclass correlation coefficient for inferior vena cava diameter was 0.86. By the second reader's measurements alone, Doppler-estimated RVSP fell from 46.2 to 18.8 mmHg and decreased in all 14 patients (median relative change −50.5%; *P* < 0.001), and the number with values of 35 mmHg or greater fell from 11 to 2 (Supplementary Table S4).

Compared with the 16 patients without paired studies, the 14 patients with paired studies had a higher estimated right atrial pressure (median 8 versus 3 mmHg; standardized mean difference +0.99; *P* = 0.014) and a larger inferior vena cava, whereas the tricuspid regurgitant pressure gradient (standardized mean difference +0.19) and signal-quality grade were similar; the median Doppler-estimated RVSP was 46.6 versus 42.7 mmHg (standardized mean difference +0.48; *P* = 0.124). Several other standardized differences were 0.4 or greater without reaching statistical significance: patients with paired studies were younger, more often male, more often had chronic lung disease, and had a slower ventricular rate. NT-proBNP was numerically higher in the paired cohort (median 2190 versus 772 pg/mL; *P* = 0.49), whereas E/e′ and left atrial volume index were similar (Supplementary Table S3). Doppler-estimated RVSP decreased in all three paired patients whose baseline value was below 35 mmHg.

Between the paired studies, six patients (43%) received intravenous loop diuretic (median cumulative furosemide-equivalent dose among recipients, 50 mg), one received isoproterenol, and one had a reduction in oral diuretic; no patient received intravenous vasodilators or renal replacement therapy, and no rate-slowing drug was discontinued. Temporary ventricular pacing was used between the studies in eight patients; in five, it was limited to the day of permanent pacemaker implantation (Supplementary Table S5). Among the seven patients who received no intravenous loop diuretic or chronotrope and had no change in oral diuretic therapy, Doppler-estimated RVSP fell in all seven (median relative change −49.6%). The median change in the tricuspid regurgitant pressure gradient was −14.7 mmHg in patients who received intravenous diuretic and −13.0 mmHg in those who did not, whereas the corresponding change in the estimated right atrial pressure was −9.5 and −5.0 mmHg; these comparisons are descriptive.

## 4 Discussion

In this single-center retrospective cohort, complete atrioventricular block was frequently accompanied by an elevated Doppler-estimated right-sided pressure phenotype that improved consistently after atrioventricular-synchronous pacing, but our design cannot establish that restoration of atrioventricular synchrony directly lowers pulmonary artery pressure. Doppler-estimated right ventricular systolic pressure was 35 mmHg or greater in 21 of 30 patients during native complete atrioventricular block, and in the 14 patients with paired studies it fell in every patient within 72 h of pacing, from a median of 46.6 to 25.0 mmHg. The fall involved both components of the estimate, and tricuspid regurgitation grade decreased in 7 patients without increasing in any. Over the same interval, however, tricuspid regurgitant signal quality tended to decline, and in the one patient with same-day invasive measurements during pacing the Doppler estimate substantially underestimated the invasive systolic pulmonary artery pressure. These observations shape both what our findings support and what they cannot.

### 4.1 Potential physiological interpretations

Several mechanisms may contribute to the observed phenotype, together with concurrent changes in loading conditions, and our data do not allow these effects to be separated. The first is loss of atrioventricular synchrony itself. When atrial and ventricular activity are dissociated, atrial contraction intermittently occurs against closed atrioventricular valves, producing cannon a waves and transient elevations in atrial and systemic venous pressure that have been recognized in pacing hemodynamic studies for decades (3, 4). In our cohort the ratio of ventricular to atrial cycle length, which indexes the degree of rate disparity rather than the presence of dissociation, was correlated with the estimated right atrial pressure, and the estimated right atrial pressure fell from 8 to 3 mmHg after pacing. This is consistent with, but does not demonstrate, a contribution from atrioventricular dissociation. Decongestive treatment given between the studies may also have contributed to the fall in the right atrial pressure component.

The second is bradycardia itself. Ventricular rate correction has been associated with a stepwise fall in invasively measured pulmonary artery pressure in bradycardia-associated pulmonary hypertension (14), and in one of our catheterized patients rate correction by temporary VVI pacing lowered both the wedge and mean pulmonary artery pressures. Although the change in heart rate was not correlated with the change in Doppler-estimated right ventricular systolic pressure in the paired cohort, 14 paired observations provide limited power, and a rate-related contribution cannot be excluded.

The third is a flow-mediated effect. Compensatory augmentation of stroke volume during bradycardia increases the volume ejected per beat through the pulmonary circulation, and this mechanism was proposed in an earlier case report of transiently elevated estimated pulmonary pressure in complete heart block (5). Our data are compatible with such a contribution: left ventricular stroke volume fell from 104.0 to 69.0 mL after pacing while cardiac output did not fall, indicating that the pre-pacing state was characterized by a low-rate, high-stroke-volume circulation that pacing reversed. Because paired outflow-tract measurements were available in only 7 patients and right-sided outflow measurements were too sparse to analyze, we regard this as a plausible contributor rather than a tested mechanism.

Concurrent changes in loading conditions must also be considered. Six of the 14 paired patients received intravenous loop diuretic between the studies, and the decrease in the estimated right atrial pressure was larger in these patients, whereas a reduction in the tricuspid regurgitant pressure gradient was also observed in patients who did not receive it. These small, nonrandomized comparisons cannot separate pacing-related effects from decongestion. The paired cohort also had a higher baseline estimated right atrial pressure than patients without paired studies, so regression toward the mean may have contributed to the decrease in that component.

One competing explanation can be addressed more directly. Ventricular pacing changes ventricular activation as well as atrioventricular timing, raising the possibility that the improvement reflected altered ventricular activation rather than restored atrioventricular synchrony. In our cohort, however, the reduction in Doppler-estimated right ventricular systolic pressure was not accompanied by narrower ventricular activation; paced QRS duration increased from a median of 129 to 170 ms and was wider in 28 of the 30 patients. This makes narrowing of ventricular electrical activation an unlikely explanation for the observed Doppler change, but does not exclude changes in mechanical synchrony, because QRS duration is not a direct measure of mechanical dyssynchrony and acute pacing studies have shown that electrical and mechanical indices of ventricular synchrony do not always change in parallel (15).

### 4.2 Relation to previous reports

The hemodynamic importance of atrioventricular synchrony has been recognized since early pacing studies, in which atrial-triggered pacing produced higher cardiac output and lower left ventricular filling pressures than rate-matched fixed-rate ventricular pacing in complete heart block (1), and later work confirmed the dependence of stroke volume and stroke work on the programmed atrioventricular interval (16). A recent randomized within-patient comparison of atrioventricular-synchronous and atrioventricular-asynchronous pacing found a higher stroke volume in every patient during synchronous pacing (2). The pulmonary pressure response, however, has been studied far less consistently; in critically ill patients, preserving atrioventricular synchrony improved systemic hemodynamics without a significant change in mean pulmonary artery pressure or pulmonary vascular resistance (17).

Elevated Doppler-estimated pulmonary pressure in complete atrioventricular block has been described in individual adult case reports, but its prevalence in an adult cohort, its consistency across patients, and its early response to atrioventricular-synchronous pacing have not been systematically characterized. A 2020 case report described a marked elevation in Doppler-estimated right ventricular systolic pressure that normalized after permanent pacing and attributed it primarily to increased right-sided stroke volume during bradycardia (5), and a recent case report described a markedly elevated Doppler-estimated pulmonary artery systolic pressure that fell rapidly after dual-chamber pacing and attributed it to atrioventricular dyssynchrony (6). Our cohort extends these single-patient observations by showing that the phenotype is common rather than exceptional and that the direction of change after pacing is uniform. It also tempers them: whereas the recent report attributes the phenomenon to atrioventricular dyssynchrony, our data support a more cautious interpretation, because Doppler signal quality changed over the same interval and invasive pressure did not invariably track the Doppler estimate.

### 4.3 Magnitude, signal quality, and Doppler–invasive discordance

The magnitude of the observed reduction should be interpreted cautiously, for two reasons that point in the same direction. Tricuspid regurgitant signal quality deteriorated in half of the paired cohort, and in Case 4 the Doppler estimate obtained during VVI pacing underestimated the same-day invasive systolic pulmonary artery pressure by a wide margin, coinciding with a fall in signal quality. Both observations raise the possibility that Doppler underestimation in the paced state exaggerated the apparent reduction. Importantly, however, Doppler-estimated right ventricular systolic pressure still decreased in all 7 patients whose signal quality was unchanged or improved, with a median relative change of −33.8%. The magnitude of the reduction is therefore uncertain and may be smaller than the 51.4% observed in the full paired cohort, whereas its direction appears less dependent on signal-quality deterioration.

The discordance itself is informative. Doppler estimation of pulmonary artery pressure is known to have wide limits of agreement against invasive measurement, with errors in both directions (7–9), and our four catheterized patients showed the same bidirectional pattern. What our serial observations add is that the direction and size of the error may vary within the same patient across rhythm and pacing states. In the native state the Doppler estimate closely matched the invasive value, whereas during VVI pacing on the same admission it did not; discrepancies of this size have been described even in simultaneous Doppler–catheterization studies (11). Beat selection is an unlikely explanation, because peak velocities were taken from the highest-velocity beat with an adequate envelope while invasive pressures were taken from a representative beat closest to the median of the automated values, an asymmetry that would bias the Doppler estimate upward rather than downward.

### 4.4 Clinical implications

Dyspnea was the most common presenting symptom in this cohort, occurring in 18 of 30 patients, so the abnormalities we describe occurred in a clinically symptomatic population; our data do not, however, establish that the Doppler findings explained the symptoms. The practical implication is interpretive. An isolated elevation in Doppler-estimated right ventricular systolic pressure discovered during untreated complete atrioventricular block should therefore be interpreted cautiously and, when clinically appropriate, reassessed after rhythm and pacing status have stabilized before it is attributed to fixed pulmonary vascular disease, for which current definitions require invasive confirmation (18). Conversely, a low Doppler estimate obtained during ventricular pacing should not by itself be considered reassuring, since the estimate underestimated the invasive pressure in the one patient in whom the two were measured on the same day. Where the distinction matters clinically, invasive assessment in a defined pacing state remains the reference.

### 4.5 Limitations

This was a retrospective, single-center study without a control group; every patient in the native cohort had complete atrioventricular dissociation, so the design cannot test an association between dissociation and the phenotype and can only describe within-patient change after pacing. The primary endpoint is a Doppler estimate rather than a measured pressure, and no patient underwent invasive measurement after atrioventricular-synchronous pacing, so we cannot state that pulmonary artery pressure itself fell. Because the pacing state is evident from the images and the electrocardiographic tracing, blinding to pacing state was not feasible for either reader. Primary measurements were made by a single investigator; an independent second reader re-read all paired studies and reproduced the direction of change, although agreement for peak tricuspid regurgitant velocity was lower in the paced state. The three-beat sensitivity analysis was feasible mainly in patients whose signal quality was preserved, and therefore could not assess beat selection in recordings whose envelope deteriorated after pacing.

Repeat echocardiography after implantation was obtained at the discretion of the treating physician rather than by protocol, and patients with paired studies had a higher estimated right atrial pressure and a slower ventricular rate at baseline than those without; regression toward the mean may therefore have contributed to the decrease in the right atrial pressure component, and the paired findings may not generalize to all patients with complete atrioventricular block. Loading conditions also changed between the studies: six of the 14 paired patients received intravenous loop diuretic. Follow-up body weight was recorded in only a minority of patients, intravenous fluid volumes could not be reliably ascertained, and blood pressure at the time of each paired echocardiographic study was not collected. The tricuspid regurgitant pressure gradient also decreased in patients who received no decongestive or chronotropic treatment, but these small, nonrandomized comparisons cannot exclude a contribution of loading changes, and the observed change cannot be attributed to pacing alone.

Programmed atrioventricular delay values were not recorded and could not be retrieved retrospectively, so the paced atrioventricular interval could not be compared with physiological timing. One patient received a single-component leadless device providing VDD pacing, which relies on mechanical rather than electrical atrial sensing; device-based atrial tracking percentages were not available for review. Atrial tracking was nonetheless confirmed electrocardiographically in all patients and, where post-implantation echocardiography was available, additionally on transmitral Doppler.

Systemic arterial oxygen saturation was obtained by pulse oximetry rather than by direct sampling in the two atrial fibrillation comparators. Pulse oximetry tends to exceed measured arterial saturation slightly, which would widen the calculated arteriovenous oxygen difference and bias the derived cardiac output downward; the cardiac outputs in these two patients were nonetheless the highest in the series, so this bias does not affect the direction of the comparison.

Right-sided outflow-tract flow measurements were available in too few patients to test the flow-mediated hypothesis directly. Diastolic tricuspid regurgitation, recently proposed as a potential marker of atrioventricular dyssynchrony in this setting (6), was not prespecified or systematically assessed and therefore could not be evaluated at the cohort level. We did not assess right ventricular strain or the pattern of right heart remodeling, both of which carry prognostic information in pulmonary vascular disease and might have provided complementary information on right ventricular function and remodeling phenotype (19, 20). The paired cohort comprised 14 patients and the invasive and serial observations involved single cases, so all mechanistic statements are hypothesis-generating. Finally, the interval between paired studies was short by design, and whether the observed changes persist beyond the first days after implantation is unknown.

### 4.6 Conclusion

Complete atrioventricular block was frequently accompanied by an elevated Doppler-estimated right ventricular systolic pressure, which decreased in every patient within 72 h of atrioventricular-synchronous pacing. The observed change is compatible with contributions from restoration of atrioventricular synchrony, correction of bradycardia, altered stroke volume, and concurrent changes in loading conditions, none of which can be separated by the present observational design. The absence of a detectable relation between the change in heart rate and the change in Doppler-estimated right ventricular systolic pressure in 14 paired observations does not exclude a rate-related mechanism, and the design cannot establish that pulmonary artery pressure itself fell. Because Doppler signal quality changed over the same interval and invasive pressure did not invariably track the Doppler estimate, an isolated elevated Doppler estimate during untreated complete atrioventricular block should be interpreted in the context of rhythm and pacing state. Prospective studies with invasive measurement in defined pacing states are needed.

## Data Availability

The datasets generated and analyzed during the current study are not publicly available because they contain information that could compromise the privacy of individual patients. De-identified data are available from the corresponding author on reasonable request, subject to approval by the Institutional Review Board of Dongkang Medical Center.
