# Supplementary material for: Elevated Doppler-estimated right ventricular systolic pressure in complete atrioventricular block and its response to atrioventricular-synchronous pacing
Source: Front Cardiovasc Med. 2026 Oct 28;13:1967085. doi: 10.3389/fcvm.2026.1967085 (PMC13751412; doi:10.3389/fcvm.2026.1967085)
Supplement: Supplementary file 1 [file Datasheet1.docx]

**Supplementary Material**

**Supplementary Tables**

**Supplementary Table 1.** Spearman correlations between baseline electrocardiographic rate parameters and the components of the Doppler-estimated right ventricular systolic pressure in the native cohort (n = 30).

| **Electrocardiographic parameter** | **ρ vs TR gradient** | **P** | **ρ vs estimated RAP** | **P** | **ρ vs Doppler RVSP** | **P** |
| --- | --- | --- | --- | --- | --- | --- |
| Ventricular rate | −0.014 | 0.943 | −0.214 | 0.256 | −0.104 | 0.584 |
| Atrial rate | +0.227 | 0.228 | +0.289 | 0.122 | +0.302 | 0.105 |
| Ventricular-to-atrial cycle-length ratio (RR/PP) | +0.236 | 0.210 | +0.580 | <0.001 | +0.428 | 0.018 |

The RR/PP ratio is the ventricular cycle length divided by the atrial cycle length, and therefore expresses the average number of atrial cycles occurring per ventricular cycle. It was evaluated as an exploratory index of the disparity between atrial and ventricular rates. It was not prespecified in the ethics-approved protocol, and no adjustment for multiple comparisons was applied; all correlations are therefore considered exploratory. Because every patient in this cohort had complete atrioventricular dissociation, the ratio indexes the average number of atrial cycles per ventricular cycle rather than the presence or extent of dissociation. Reciprocal parameters (RR interval, PP interval) are omitted because they carry the same information as the corresponding rates. The ratio showed a stronger correlation with the estimated right atrial pressure than with the tricuspid regurgitant pressure gradient, suggesting that its association with Doppler-estimated RVSP may largely reflect the estimated right atrial pressure component; the two coefficients were not compared formally. The ratio was correlated with inferior vena cava diameter to a similar degree (ρ = +0.552, P = 0.002). RAP, right atrial pressure; RVSP, right ventricular systolic pressure; TR, tricuspid regurgitant.

**Supplementary Table 2.** Post hoc sensitivity analyses excluding patients with aortic stenosis — all mild — patients with chronic lung disease, and patients in whom tricuspid regurgitant signal quality deteriorated after pacing.

| **Analysis** | **Full cohort**  **(native n = 30;**  **paired n = 14)** | **Aortic stenosis excluded**  **(native n = 24;**  **paired n = 12)** | **Chronic lung disease excluded**  **(native n = 25;**  **paired n = 10)** | **TR signal quality unchanged or improved**  **(native n = —;**  **paired n = 7)** |
| --- | --- | --- | --- | --- |
| RR/PP ratio vs Doppler-estimated RVSP | ρ = +0.428, P = 0.018 | ρ = +0.432, P = 0.035 | ρ = +0.374, P = 0.065 | — |
| RR/PP ratio vs estimated RAP | ρ = +0.580, P < 0.001 | ρ = +0.551, P = 0.005 | ρ = +0.571, P = 0.003 | — |
| Elevated Doppler-estimated RVSP (≥35 mmHg), n (%) | 21/30 (70.0) | 17/24 (70.8) | 17/25 (68.0) | — |
| Paired Doppler-estimated RVSP, native → paced, mmHg | 46.6 → 25.0, P < 0.001 | 46.3 → 22.3, P < 0.001 | 45.5 → 22.3, P = 0.002 | 36.2 → 16.1, P = 0.016 |
| Median relative change in Doppler-estimated RVSP | −51.4% | −51.4% | −46.7% | −33.8% |
| Patients with a reduction in Doppler-estimated RVSP | 14/14 | 12/12 | 10/10 | 7/7 |

Aortic stenosis status was based on the clinical echocardiographic assessment; all recorded cases were mild. Patients with a planimetered aortic valve area below 1.5 cm² were excluded during cohort selection. Chronic lung disease included diagnoses of asthma, chronic obstructive pulmonary disease, emphysema, and bronchiectasis. The tricuspid regurgitant signal-quality subgroup comprises patients whose signal-quality grade was unchanged or improved after pacing; because it is defined by a change between the paired studies, it exists only within the paired cohort, and the native-cohort analyses are therefore not applicable (—). For the primary paired endpoint no estimate changed materially and the direction and statistical significance were preserved in all three sensitivity analyses. The relative reduction was smaller in the signal-quality subgroup, indicating that deterioration in envelope quality may have accentuated the magnitude of the fall observed in the full cohort; the direction and statistical significance were nonetheless preserved. The exploratory association between the RR/PP ratio and Doppler-estimated RVSP was no longer significant after exclusion of patients with chronic lung disease, whereas its association with the estimated right atrial pressure persisted; this association should therefore be regarded as hypothesis-generating. Correlations are Spearman rank coefficients and paired comparisons use the Wilcoxon signed-rank test. RAP, right atrial pressure; RVSP, right ventricular systolic pressure; TR, tricuspid regurgitant.

**Supplementary Table 3.** Baseline characteristics of patients with and without paired post-implantation echocardiography within the native cohort (n = 30).

| **Variable** | **Paired (n = 14)** | **Not paired (n = 16)** | **SMD** | **P** |
| --- | --- | --- | --- | --- |
| Age, years | 77.5 (69.3–81.8) | 80.5 (74.5–85.0) | −0.55 | 0.288 |
| Male sex, n (%) | 9 (64.3) | 6 (37.5) | +0.56 | 0.272 |
| Dyspnea as presenting symptom, n (%) | 7 (50.0) | 11 (68.8) | −0.39 | 0.457 |
| Chronic lung disease, n (%) | 4 (28.6) | 1 (6.3) | +0.62 | 0.157 |
| Ventricular rate on electrocardiogram, beats/min | 35 (33–38) | 41 (37–44) | −0.40 | 0.091 |
| Peak tricuspid regurgitant velocity, m/s | 3.06 (2.58–3.48) | 3.00 (2.49–3.13) | +0.21 | 0.454 |
| Tricuspid regurgitant pressure gradient, mmHg | 37.5 (26.6–48.5) | 36.0 (24.7–39.1) | +0.19 | 0.454 |
| Inferior vena cava diameter, cm | 2.01 (1.80–2.22) | 1.67 (1.46–2.01) | +0.53 | 0.109 |
| Estimated right atrial pressure, mmHg | 8 (8–15) | 3 (3–8) | +0.99 | 0.014 |
| Doppler-estimated RVSP, mmHg | 46.6 (37.5–59.2) | 42.7 (30.9–44.4) | +0.48 | 0.124 |
| Doppler-estimated RVSP ≥35 mmHg, n (%) | 11 (78.6) | 10 (62.5) | +0.36 | 0.440 |
| Tricuspid regurgitation grade | 1 (0–1) | 1 (0–1) | +0.24 | 0.484 |
| Tricuspid regurgitant signal-quality grade | 3 (2–3) | 3 (2–3) | −0.10 | 0.889 |
| E/e′ | 17.0 (11.3–21.2) | 16.5 (13.1–19.2) | +0.10 | 0.884 |
| Left atrial volume index, mL/m²* | 44.4 (39.4–53.7) | 50.9 (41.9–56.5) | −0.33 | 0.510 |
| NT-proBNP, pg/mL† | 2190 (743–3500) | 772 (440–3141) | +0.29‡ | 0.490 |
| Left ventricular ejection fraction, % | 64.0 (62.5–65.0) | 64.5 (61.3–66.3) | +0.10 | 0.950 |

Values are median (interquartile range) or n (%). Post-implantation echocardiography was not mandated by protocol and was obtained at the discretion of the treating physician. The standardized mean difference (SMD) is the difference between the paired and not-paired groups divided by the pooled standard deviation, so a positive value indicates a higher value in the paired cohort; for categorical variables it was calculated from the proportions. P values are from the Mann–Whitney U test for continuous and ordinal variables and from Fisher's exact test for categorical variables. This comparison was performed post hoc in response to peer review, involves 17 variables in 30 patients, and was not adjusted for multiple comparisons; it is therefore descriptive. The ventricular rate is from the baseline electrocardiogram and differs from the heart rate during echocardiography reported in Table 3. *Available in 12 paired and 15 not-paired patients. †Available in 13 paired and 15 not-paired patients. ‡Calculated after logarithmic transformation. NT-proBNP, N-terminal pro-B-type natriuretic peptide; RVSP, right ventricular systolic pressure; SMD, standardized mean difference.

**Supplementary Table 4.** Post hoc measurement analyses in the paired cohort (n = 14): three-beat averaging sensitivity analysis and independent second-reader assessment.

| **Measure** | **Native complete AV block** | **AV-synchronous pacing** | **Result / all studies** | **P** |
| --- | --- | --- | --- | --- |
| ***Part A. Three-beat averaging sensitivity analysis (primary reader)*** | | | | |
| Studies with three consecutive identifiable peaks | 9/14 | 10/14 | Complete pairs, 6/14 | — |
| Signal-quality change in complete pairs (worsened / unchanged / improved) | — | — | 1 / 4 / 1 (all pairs, 7 / 5 / 2) | — |
| Highest-beat minus three-beat mean velocity, m/s (complete pairs) | 0.18 (0.07–0.29) | 0.12 (0.05–0.24) | — | 0.56 |
| Peak tricuspid regurgitant velocity, three-beat mean, m/s | 2.99 (2.38–3.31) | 2.37 (1.81–3.02) | Decreased in 5/6 | 0.063 |
| Tricuspid regurgitant pressure gradient, three-beat mean, mmHg | 35.8 (22.9–44.0) | 23.3 (13.1–36.7) | Decreased in 5/6 | 0.063 |
| Doppler-estimated RVSP, three-beat mean, mmHg | 46.9 (36.2–55.1) | 26.3 (16.1–39.7) | Decreased in 6/6; −42.7% (−59.9 to −26.4) | 0.031 |
| Doppler-estimated RVSP, primary method, same six patients, mmHg | 51.2 (38.4–60.3) | 28.5 (17.2–45.2) | Decreased in 6/6; −40.8% (−61.8 to −21.2) | 0.031 |
| ***Part B. Independent second reader (all 28 studies from the 14 paired patients)*** | | | | |
| Peak tricuspid regurgitant velocity, ICC (95% CI) | 0.984 (0.952–0.995) | 0.825 (0.430–0.945) | — | — |
| Peak tricuspid regurgitant velocity, bias (limits of agreement), m/s | −0.03 (−0.22 to +0.15) | −0.24 (−0.92 to +0.43) | — | — |
| Signal-quality grade, weighted κ (95% CI); exact agreement | — | — | 0.84 (0.68–0.96); 24/28 | — |
| Inferior vena cava diameter, ICC (95% CI); bias (limits of agreement), cm | — | — | 0.86 (0.73–0.93); −0.03 (−0.47 to +0.41) | — |
| Estimated right atrial pressure category, weighted κ (95% CI); exact agreement | — | — | 0.87 (0.72–1.00); 25/28 | — |
| Second-reader peak tricuspid regurgitant velocity, m/s | 3.07 (2.46–3.42) | 1.97 (1.64–2.36) | Decreased in 14/14 | <0.001 |
| Second-reader Doppler-estimated RVSP, mmHg | 46.2 (38.7–56.8) | 18.8 (13.7–25.3) | Decreased in 14/14; −50.5% | <0.001 |
| Second-reader Doppler-estimated RVSP ≥35 mmHg, n | 11 | 2 | — | — |

Values are median (interquartile range) unless stated otherwise. Part A: the primary reader re-measured peak tricuspid regurgitant velocity in the first three consecutive beats with an identifiable peak, counted from the start of the same continuous-wave recording used for the primary measurement; the three velocities were averaged before the pressure gradient was calculated, and the original estimated right atrial pressure was retained. Recordings without three consecutive identifiable peaks were treated as missing. This analysis was defined before re-measurement and is reported as a sensitivity analysis; the primary analysis is unchanged. Relative changes are medians of patient-level relative changes. Part B: an independent echocardiographer, unaware of the primary measurements and the clinical reports, measured peak tricuspid regurgitant velocity by the primary beat-selection rule, graded signal quality, and measured the inferior vena cava in all 28 studies presented in randomized order; patient identifiers were retained for file handling, the reader was instructed not to compare the paired study from the same patient, and pacing state could not be concealed. In Part B the columns give the native-state and paced-state values, and the third column gives agreement across all 28 studies or the paired result; agreement for peak tricuspid regurgitant velocity is reported separately by pacing state. The intraclass correlation coefficient (ICC) is the two-way random-effects, absolute-agreement, single-measure coefficient; bias is the second-reader value minus the primary-reader value, with 95% limits of agreement. κ is linearly weighted, with a patient-level cluster-bootstrap 95% CI. The second-reader Doppler-estimated RVSP was calculated entirely from the second reader's velocity and inferior vena cava measurements. Paired comparisons use the exact Wilcoxon signed-rank test. AV, atrioventricular; ICC, intraclass correlation coefficient; RVSP, right ventricular systolic pressure.

**Supplementary Table 5.** Treatments and clinical status between the paired echocardiographic studies (n = 14).

| **Variable** | **Value** |
| --- | --- |
| ***Timing*** | |
| Interval between paired studies, days | 2 (1–3); range 1–8 |
| Implantation to follow-up study, days | 1 (1–1); range 0–3 |
| Temporary ventricular pacing between studies | 8 (57.1); limited to the day of implantation in 5 |
| ***Treatment between studies*** | |
| Oral loop diuretic at the baseline study | 1 (7.1) |
| Intravenous loop diuretic | 6 (42.9) |
| Cumulative furosemide-equivalent dose among recipients, mg | 50 (40–60) |
| Oral diuretic started or increased / reduced | 0 / 1 (7.1) |
| Intravenous isoproterenol | 1 (7.1) |
| Intravenous vasodilator | 0 |
| Renal replacement therapy | 0 |
| Rate-slowing drug discontinued | 0 |
| ***Clinical status*** | |
| Supplemental oxygen at the baseline / follow-up study | 5 (35.7) / 4 (28.6) |
| Creatinine at baseline / follow-up, mg/dL | 1.02 (0.76–1.29) / 0.89 (0.78–0.99) |
| Change in creatinine, mg/dL | −0.09 (−0.31 to +0.10) |
| Pulmonary congestion on chest radiograph at baseline | 4 (28.6); improved at follow-up in 3 |
| ***Change in Doppler-estimated RVSP by treatment*** | |
| Intravenous loop diuretic (n = 6): RVSP / TR gradient / estimated RAP | −54.3% / −14.7 mmHg / −9.5 mmHg; decreased in 6/6 |
| No intravenous loop diuretic (n = 8): RVSP / TR gradient / estimated RAP | −42.4% / −13.0 mmHg / −5.0 mmHg; decreased in 8/8 |
| No intravenous loop diuretic, chronotrope, or change in oral diuretic (n = 7): RVSP | −49.6%; decreased in 7/7 |

Values are median (interquartile range) or n (%). Treatments refer to the interval between the baseline (native) study and the follow-up study after atrioventricular-synchronous pacing. Intravenous loop diuretic doses are expressed as furosemide equivalents (furosemide 40 mg = bumetanide 1 mg = torsemide 20 mg). Changes by treatment are medians of patient-level changes (relative change for RVSP, absolute change for the TR gradient and estimated RAP); these comparisons are descriptive, the groups are small and nonrandomized, and no statistical test was applied. Body weight at follow-up and intravenous fluid volumes were not reliably available, and blood pressure at each paired echocardiographic study was not collected; these are not reported. RAP, right atrial pressure; RVSP, right ventricular systolic pressure; TR, tricuspid regurgitant.

**Supplementary Figure legends**

**Supplementary Figure 1.** Representative Doppler tracings illustrating tricuspid regurgitant signal-quality grading and atrioventricular dissociation. (A–C) Continuous-wave tricuspid regurgitant recordings graded 1 to 3 on the completeness of the envelope and the identifiability of its peak: grade 1, an incomplete envelope whose peak could be identified only with reduced confidence; grade 2, a partially complete envelope with an identifiable peak; grade 3, a complete envelope with an unambiguous peak. Peak velocity was measurable in all recordings, including those graded 1, and beat-to-beat variation in velocity was not used as a grading criterion because it reflects the physiology of atrioventricular dissociation rather than measurement error. (D–F) Transmitral pulsed-wave Doppler in Case 4 during native complete atrioventricular block, temporary VVI pacing, and atrioventricular-synchronous pacing. During native block, A waves recur at intervals unrelated to ventricular diastole. VVI pacing regularizes the ventricular rate but does not track atrial activity, so dissociation persists and the A waves that appear within diastole are dissociated atrial contractions rather than evidence of synchrony. After atrioventricular-synchronous pacing, the E and A waves recur in a fixed sequence within each cardiac cycle. AV, atrioventricular; DDD, dual-chamber pacing; TR, tricuspid regurgitant; VVI, ventricular-inhibited pacing.

**Supplementary Figure 2.** Serial Doppler and invasive pulmonary artery observations across pacing states in Case 4. Panels (A–D) show native complete atrioventricular block, temporary VVI pacing on day 1, temporary VVI pacing on day 8, and atrioventricular-synchronous pacing. The upper row shows the continuous-wave tricuspid regurgitant envelope with its peak velocity, signal-quality grade, and the resulting Doppler-estimated right ventricular systolic pressure; the lower row shows the corresponding invasive pulmonary artery tracing. At each state with invasive data, Doppler and invasive measurements were obtained on the same day but were not acquired simultaneously. The tricuspid regurgitant envelope was complete during native block (grade 3) and became progressively less complete during pacing (grades 2 and 1), and the Doppler estimate fell from 57.3 to 24.2 mmHg between the native state and day 1 of VVI pacing while the invasive systolic pulmonary artery pressure fell only from 60 to 52 mmHg and the mean pressure rose from 38 to 43 mmHg. Systemic arterial pressure displayed on the monitor during the day 1 study was 253/106 mmHg. No invasive measurement was obtained during atrioventricular-synchronous pacing. Values printed on the images are the on-screen output of the recording systems. mPAP, mean pulmonary artery pressure; PAP, pulmonary artery pressure; RVSP, right ventricular systolic pressure; TR, tricuspid regurgitant; VVI, ventricular-inhibited pacing.
